# Metabolic and Nutritional Disorders Following the Administration of Immune Checkpoint Inhibitors: A Pharmacovigilance Study

**DOI:** 10.3389/fendo.2021.809063

**Published:** 2022-01-25

**Authors:** Yinghong Zhai, Xiaofei Ye, Fangyuan Hu, Jinfang Xu, Xiaojing Guo, Xiang Zhou, Yi Zheng, Xinxin Zhao, Xiao Xu, Yang Cao, Jia He

**Affiliations:** ^1^ Tongji University School of Medicine, Shanghai, China; ^2^ Department of Health Statistics, Second Military Medical University, Shanghai, China; ^3^ Department of Medical Service, Naval Hospital of Eastern Theater Zhoushan, Zhejiang, China; ^4^ Clinical Epidemiology and Biostatistics, School of Medical Sciences, Örebro University, Örebro, Sweden; ^5^ Unit of Integrative Epidemiology, Institute of Environmental Medicine, Karolinska Institutet, Stockholm, Sweden

**Keywords:** metabolic and nutritional disorders, immune checkpoint inhibitors, FAERS database, pharmacovigilance study, disproportionality analysis, reporting odds ratio, information component

## Abstract

**Background:**

Although several metabolic and nutritional disorders (MNDs) have been reported in the recipients of immune checkpoint inhibitors (ICIs), these events have not been fully captured and comprehensively characterized in real-world population.

**Objectives:**

To provide complete metabolic and nutritional toxicity profiles after ICIs (single and combined) initiation through an integrated big database.

**Methods:**

Reporting odds ratios (ROR) and information component (IC) based on statistical shrinkage transformation were utilized to perform disproportionality analysis using the US Food and Drug Administration Adverse Events Reporting System. Both ROR and IC were used to calculate disproportionality when compared with the whole database, but only ROR was used when comparison was made for different ICI strategies. Only when both the lower limits of 95% confidence intervals (CIs) for ROR (ROR_025_) and IC (IC_025_) exceeded specified threshold values (1 and 0, respectively) was regarded as a signal.

**Results:**

A total of 29,294,335 records were involved and 8,662 records were for MNDs in patients exposed to ICIs. Statistically significant association was detected between ICIs use and total MNDs (IC_025_/ROR_025 _= 1.06/2.19). For monotherapy, three ICI monotherapies (anti-PD-1, anti-PDL-1, and anti-CTLA-4) were all disproportionately associated with MNDs. Statistically significant differences in reporting frequencies also emerged when comparing anti-PD-1 with anti-PD-L1/anti-CTLA-4 monotherapy, with RORs of 1.11 (95%CI 1.01-1.21), and 1.35 (95%CI 1.23-1.48), respectively. Notably, combination therapy was associated with a higher reporting frequency of theses toxicities compared to monotherapy with a ROR of 1.56 (95%CI 1.48-1.64). Additionally, disproportionality analysis at High-level Group Term level highlighted eight broad entities of MNDs. Further disproportionality analysis at Preferred Term level indicated a wide range and varied strength of signals. For ICI monotherapy, nivolumab and pembrolizumab showed the broadest spectrum of MNDs. For combination therapy, a variety of signals were detected for nivolumab + ipilimumab therapy even comparable to two PD-1 monotherapies.

**Conclusion:**

Metabolic and nutritional complications could be provoked by ICI monotherapy (especially anti-PD-1) and further reinforced by combination therapy. Clinicians and patients should be informed about these potential risks that might be encountered in real-world practice. Aforehand education and regular monitoring of related biochemical parameters (calcium, sodium, potassium, protein) are recommended to ensure better cancer survivorship.

## Introduction

Immune checkpoint inhibitors (ICIs) are a new type of immunotherapy drug that is originally used to improve survival in metastatic melanoma patients (1). Over the past few years, aside from the clinical success of these therapies in patients with melanoma, substantial improvements could also be achieved in patients with other advanced-stage cancer subtypes, including small cell lung cancer, renal cell carcinoma, urothelial cancer, head and neck squamous cell carcinoma, gastric cancer, etc., representing a landmark event in cancer immunotherapy (2). ICIs medications can enhance self-immune functions against cancer cells by blocking negative regulators expressed on immune or tumour cells (3). Currently, ICIs drugs include monoclonal autoantibodies specifically target cytotoxic T lymphocyte associated antigen 4 (anti-CTLA-4), programmed death 1 (anti-PD-1) and programmed cell death ligand 1(anti-PD-L1) (4).

Despite favorable benefits, ICIs medications are also accompanied by peculiar dysimmune toxicities, referred to immune-related adverse events (irAEs), in a wide variety of organs. Although clinical trials are mandatory to establish efficacy for novel meditations, and also be efficient to detect the most frequent adverse effects of a drug, they could not reflect all situations in real-world practice because some rare and serious events could not be detected until a drug has been widely administered in clinics (5). With the number of patients exposed to ICIs on the rise, several endocrine complications after ICIs also come to the spotlight. If not recognized and treated promptly, they can lead to extremely serious consequences. Several cases of fatal or life-threatening type 1 diabetes mellitus in the recipients of ICIs have been reported (6, 7). In the previous study (8), we have characterized and compared the profiles of endocrine toxicity among various ICI strategies. Indeed, several important adverse events (AEs) such as hyponatremia, hypokalemia, hypocalcemia, and other electrolyte abnormalities have been observed in patients receiving ICIs (9–12). However, these disorders have not been comprehensively evaluated and characterized in a real-world population receiving ICIs. In the current study, we further expanded the research question to metabolic and nutritional disorders (MNDs) which spanned a vaster range of conditions, leveraging data from the U.S. FDA’s Adverse Event Reporting System (FAERS). We aimed to bring a deeper insight into this issue and our study might supplement existing literatures by systematically identifying all possible metabolic and nutritional risks associated with ICIs and prioritizing several major ones warranting further attention.

## Materials and Methods

### Data Source

The study was performed involving cumulative data available from the FAERS database collected from the first quarter of 2014 to the first quarter of 2019. FAERS is a publicly accessible database maintained by FDA for post-marketing adverse events surveillance, based on voluntary reporting from manufacturers, healthcare professionals, lawyers, and consumers. FAERS contains demographic and administrative information, drug information, reaction information, patient outcome information, and information on the source of the reports. FAERS allows for the signal detection and quantification of the association between suspected drugs and reporting of specific AEs. FAERS files are made publicly available on a quarterly basis and are accessed at the website: https://fis.fda.gov/extensions/FPD-QDE-FAERS/FPD-QDE-FAERS.html. Several studies have demonstrated the benefit of FAERS in the early detection of safety concerns, especially for newly approved medications, and also for the AEs with low incidence (13, 14).

### Study Drugs and Adverse Events

Study drugs included anti-PD-1 antibodies (nivolumab (*opdivo*), pembrolizumab (*keytruda*) and cemiplimab (*libtayo*)), anti-PD-L1 antibodies (atezolizumab (*tecentriq*), avelumab (*bavencio*) and durvalumab (*imfinzi*)), and anti-CTLA-4 antibodies (ipilimumab (*yervoy*) and tremelimumab). Both the original drug names and standard drug names were used to identify ICIs associated records.

AEs in FAERS are coded using the Medical Dictionary for Regulatory Activities (MedDRA) terminology. Each record is coded with a preferred term (PT) and a given PT can be assigned to one or more High-level Terms (HLTs), High-level Group Terms (HLGTs), and System Organ Class (SOC) levels (15). In our research, each record involving one of the PTs related to metabolic and nutritional disorders (identified with SOC code 10027433 according to MedDRA version 22.0) was considered as a metabolic and nutritional event. In addition, these events were further classified into 14 broad groups according to HLGT codes (**Table S1**).

### Statistical Analysis

In pharmacovigilance study, the term signal refers to the association within a specific drug-AE pair, which may be detected using several methods (16). Currently, the most common signal detection methods are disproportionality analysis that aim to discover higher-than-expected drug-AE pairs in the database (17). Two disproportionality algorithms, reporting odds ratios (ROR) and bayesian confidence propagation neural networks of information components (IC), were used in the current study (18, 19). In addition, the simple shrinkage transformation was leveraged to address the shortcoming that the two algorithms were sensitive to random fluctuations for rare events (20). When comparing reporting frequencies in different ICI regimens, only ROR was used to calculate disproportionality. The relevant formulas are:


$$\text{ROR}=(N_{observed}+0.5)/(N_{expected}+0.5)$$

$$\text{IC}=\log_{2}((N_{observed}+0.5)/(N_{expected}+0.5))$$

$$N_{expected}=\frac{n_{drug}*n_{event}}{n_{total}}$$

where *N_expected_* and *N_observed_* denote the number of records expected and observed for the target drug-AE pair, respectively; *N_drug_* and *N_event_* denote the total number of records for the selected drug and AE, respectively; and *N_total_* is the total number of records involved.

The two methods estimate the lower boundaries of the 95% confidence interval (CI) for the disproportionality statistics, i.e. ROR_025_ and IC_025_, respectively. The occurrence of a signal is defined as both the lower boundaries exceeding the specified thresholds, i.e. 1 and 0, respectively. After depicting the overall profile, disproportionality analysis was performed in depth at HLGT and PT levels. All statistical analyses were performed in SAS 9.4 (SAS Institute, Cary, NC).

## Results

### Baseline Characteristics of the Patients

Among a total of 29, 294, 335 records in the FAERS pharmacovigilance database included, 8662 were documented for MNDs after receiving ICIs. In the affected patients, males accounted for a larger proportion than females (57.32% vs. 33.79%). Hospitalization was the most frequently reported severe outcome (43.28%), followed by death (20.82%). Other undefined serious AEs accounted for 22.29% (**Table 1**).

**Table 1:** Clinical characteristics of patients with metabolic and nutritional complications*.

	Induced by ICIs N (%)	Induced by other drugs N (%)
**Total number**	8662 (100.00)	884267 (100.00)
**Gender**		
Male	4965 (57.32)	413252 (46.73)
Female	2927 (33.79)	327532 (37.04)
Missing	770 (8.89)	143483 (16.23)
**Age**		
<65	3266 (37.70)	334363 (37.81)
>=65	3848 (44.42)	297611 (33.66)
Missing	1548 (17.87)	252293 (28.53)
**Year**		
2014	180 (2.08)	67138 (7.59)
2015	66 (0.76)	141955 (16.05)
2016	1401 (16.17)	182787 (20.67)
2017	2207 (25.48)	187056 (21.15)
2018	3839 (44.32)	242151 (27.38)
2019Q1	969 (11.19)	63180 (7.14)
**Outcome**		
Death	1803 (20.82)	95849 (10.84)
Life-threatening	592 (6.83)	52859 (5.98)
Disability	135 (1.56)	22281 2.52%
Hospitalization	3749 (43.28)	342831 (38.77)
Congenital anomaly	0 (0.00)	1556 (0.18)
Required intervention	2 (0.02)	258 (0.03)
Other serious AEs	1931 (22.29)	221098 (25.00)
Missing	450 (5.20)	147535 (16.68)
**Report countries**		
United States	2906 (33.55)	433834 (49.06)
Japan	1999 (23.08)	54161 (6.12)
Great Britain	222 (2.56)	50992 (5.77)
Germany	359 (4.140)	28178 (3.19)
France	633 (7.31)	48971 (5.54)
Canada	166 (1.92)	30930 (3.50)
Italy	267 (3.08)	27418 (3.10)
Other countries	1141(13.17)	146603 (16.58)
Missing	969 (11.19)	63180 (7.14)

*N, the number of records.

### Disproportionality by Immunotherapy Regimens

In general, MNDs were significantly more reported in the recipient of ICIs compared to all patients in the database, with an IC_025_/ROR_025_ of 1.06/2.19 (**Table 2**). Three ICI monotherapies, anti-PD-1, anti-PDL-1, and anti-CTLA-4, were significantly associated with more reported metabolic and nutritional complications (**Table 2**). Significant differences emerged when comparing anti-PD-1 with anti-PD-L1/anti-CTLA-4 monotherapies, with RORs of 1.11 (95%CI 1.01-1.21) and 1.35 (95%CI 1.23-1.48), respectively. Remarkably, disproportionality in combination therapy was significantly larger than that in monotherapy corresponding to a ROR of 1.56 (95%CI 1.48-1.64). At HLGT level, disproportionality also appeared in eight class-specific metabolic and nutritional complications, including acid-base disorders, metabolism disorders, appetite and general nutritional disorders, diabetic complications, etc., with IC_025_/ROR_025_ ranging from 0.31/1.26 to 3.45/11.70 (**Table 3**).

**Table 2:** Disproportionality by immunotherapy regimens*.

Strategy	Drug	N	IC	IC_025_	IC_975_	ROR	ROR_025_	ROR_975_
**Total**	Total ICIs	8662	1.10	**1.06**	1.14	2.24	**2.19**	2.28
**Monotherapy**	Anti-PD-1	5375	1.02	**0.98**	1.07	2.10	**2.05**	2.16
	Nivolumab	3639	1.06	**1.00**	1.11	2.16	**2.08**	2.23
	Pembrolizumab	1732	0.95	**0.87**	1.03	2.00	**1.90**	2.10
	Cemiplimab	4	0.10	-1.80	2.00	1.08	0.40	2.92
	Anti-PD-L1	576	0.88	**0.75**	1.02	1.90	**1.74**	2.06
	Atezolizumab	430	0.94	**0.78**	1.10	1.98	**1.80**	2.19
	Avelumab	54	0.81	**0.36**	1.27	1.80	**1.37**	2.37
	Durvalumab	92	0.66	**0.31**	1.00	1.60	**1.30**	1.98
	Anti-CTLA-4	515	0.61	**0.47**	0.76	1.56	**1.42**	1.70
	Ipilimumab	509	0.60	**0.46**	0.75	1.54	**1.41**	1.69
	Tremelimumab	6	1.80	**0.31**	3.28	3.77	**1.59**	8.89
**Anti-PD-1 vs anti-PD-L1**						1.11	**1.01**	1.21
**Anti-PD-1 vs anti-CTLA-4**						1.35	**1.23**	1.48
**Polytherapy**	Polytherapy1	31	1.67	**1.06**	2.27	3.41	**2.35**	4.94
	Polytherapy2	2057	1.56	**1.49**	1.64	3.16	**3.02**	3.30
	Polytherapy3	68	1.55	**1.15**	1.96	3.12	**2.43**	4.01
**Polytherapy vs Monotherapy**						1.56	**1.48**	1.64

*Bold text denotes significant signals. Polytherapy1: Nivolumab+ pembrolizumab+ ipilimumab; Polytherapy2: Nivolumab+ ipilimumab; Polytherapy3: Pembrolizumab+ ipilimumab; N, number of records; IC_025_: the lower end of the 95% confidence interval of IC; IC_975_: the upper end of the 95% confidence interval of IC; ROR_025_: the lower end of the 95% confidence interval of ROR; ROR_975_: the upper end of the 95% confidence interval of ROR.

**Table 3:** Disproportionality for total and class-specific metabolic and nutritional complications after ICIs initiation*.

Group	N	IC	IC_025_	IC_975_	ROR	ROR_025_	ROR_975_
Acid-base disorders	738	0.72	**0.60**	0.84	1.65	**1.54**	1.78
Metabolism disorders	2293	3.52	**3.45**	3.59	12.21	**11.70**	12.74
Appetite and general nutritional disorders	1504	0.84	**0.75**	0.92	1.80	**1.71**	1.90
Diabetic complications	337	1.35	**1.17**	1.53	2.58	**2.31**	2.87
Bone, calcium, magnesium and phosphorus metabolism disorders	380	0.47	**0.31**	0.64	1.39	**1.26**	1.54
Lipid metabolism disorders	40	-1.37	-1.90	-0.84	0.39	0.28	0.53
Electrolyte and fluid balance conditions	2119	0.77	**0.70**	0.84	1.72	**1.65**	1.80
Food intolerance syndromes	10	-3.58	-4.68	-2.47	0.08	0.04	0.15
Glucose metabolism disorders	1085	1.19	**1.09**	1.29	2.30	**2.17**	2.45
Inborn errors of metabolism	1						
Iron and trace metal metabolism disorders	18	-1.42	-2.22	-0.61	0.23	0.03	1.62
Protein and amino acid metabolism disorders	83	1.50	**1.14**	1.87	2.86	**2.30**	3.55
Purine and pyrimidine metabolism disorders	43	-0.38	-0.89	0.13	0.77	0.57	1.04
Vitamin related disorders	11	-2.36	-3.41	-1.31	0.19	0.11	0.35

*N: the number of records with metabolic and nutritional AEs reported for ICIs; IC_025_: the lower boundary of the 95% confidence interval of IC; IC_975_: the upper boundary of the 95% confidence interval of IC; ROR_025_: the lower end of the 95% confidence interval of ROR; ROR_975_: the upper end of the 95% confidence interval of ROR. Both IC_025_ >0 and ROR_025_ exceeds 1 was decided a signal occurred. Bold text denotes a significant signal.

### Comparison of the Toxicity Profile in Different ICI Regimens

**Figure 1:**
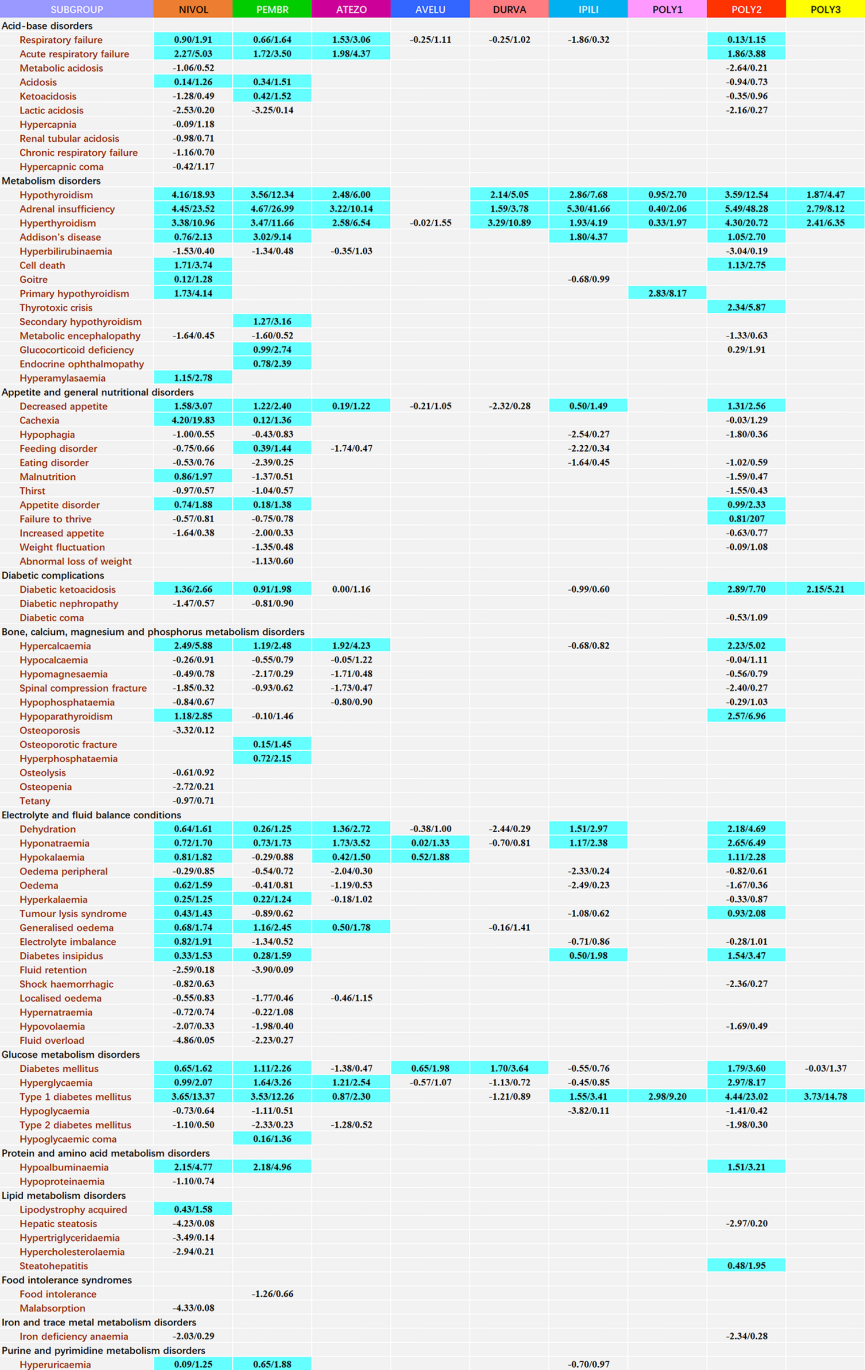
Safety profile of metabolism and nutrition diseases according to different ICI regimens*. *Each cell of the safety profile contains the values of IC_025_ and ROR_025_(lower end of the 95% confidence interval of IC and ROR, respectively); NIVOL, nivolumab; PEMBR, pembrolizumab; ATEZO, atezolizumab; AVELU, avelumab; DURVA, durvalumab; IPILI, ipilimumab; POLY1, nivolumab+ pembrolizumab+ ipilimumab; POLY2, nivolumab+ ipilimumab; POLY3, pembrolizumab+ ipilimumab. To obtain robust results and reduce the false positive signals, signal values were only calculated for complications with at least 3 records. A signal was defined as both IC_025_ >0 and ROR_025_ >1 and highlighted in blue.

For disproportionality at PT level, a wide array of signals emerged in specific ICI regimens with different occurrence frequencies (**Figure 1**). Due to the hierarchical and multiaxial nature of MedDRA, many of them overlapped with that reported in our previous article (8). For ICI monotherapy, nivolumab presented the broadest spectrum of metabolism and nutrition events, including 33 detected signals with IC_025_/ROR_025_ ranging from 0.09/1.25 for hyperuricaemia to 4.45/23.52 for adrenal insufficiency. The followed one was pembrolizumab, including 30 detected signals with IC_025_/ROR_025_ ranging from 0.12/1.36 for cachexia to 4.67/26.99 for adrenal insufficiency, which overlapped mostly with the signals detected for nivolumab. Among three PD-L1 drugs, atezolizumab was mostly associated with MNDs, with 13 detected signals and IC_025_/ROR_025_ ranging from 0.19/1.22 for decreased appetite to 3.22/10.14 for adrenal insufficiency. A similar trend was observed for ipilimumab, with a total of 9 signals detected and IC_025_/ROR_025_ ranging from 0.50/1.49 for decreased appetite to 5.30/41.66 for adrenal insufficiency. For combination therapy, remarkably, a total of 24 signals were detected for nivolumab+ ipilimumab therapy, with IC_025_/ROR_025_ ranging from 0.13/1.15 for respiratory failure to 5.49/48.28 for adrenal insufficiency.

## Discussion

The advent of ICI Immunotherapy has revolutionized the treatment of cancer therapy during the past decades (21). Impressive single-agent activity of various ICI medications has been proved in a broad spectrum of solid tumor indications (22). In addition, to enhance antitumor immune responses, combination therapy of ICIs (such as ipilimumab + nivolumab) has also been explored and approved effective for several cancers (23). Several clinical trials on novel ICI drugs and ICI combination strategies are also undergoing (24, 25). Whereas, successful ICI therapies should not only improve survival outcomes but also minimize the toxic effects induced by them. Among irAEs caused by ICIs, most metabolic and nutritional complications associated with ICIs are mild. However, with the extensive use of ICIs in a continuously increasing number of patients, some rare side effects emerge, which have not been reported in previous clinical trials. Despite several articles have mentioned this issue, they are case reports or retrospective studies limited to relatively small populations and did not provide a comprehensive analysis of metabolic and nutritional toxicity. It has been reported that ICI medications act through distinct immunologic mechanisms thus should not be treated as one entity (3). Herein, we conducted a real-world study to explore and characterize the metabolic and nutritional toxicity profiles of different ICI drugs and ICI combinations based on the FAERS database. The main strength of our study is the enormous real-world records involved that allow us to explore the question more in depth compared to the existing studies.

In general, our findings supported a significant association between MNDs and ICI drugs (alone or in combination) and combination therapies showed significant higher reporting frequencies compared with monotherapies. Two previous pharmacovigilance studies based on Vigibase reported higher chance of developing cardiovascular toxicities (26) and neurologic toxicities (27) associated with combination immunotherapies in the patients receiving ICIs. Our findings suggested that it may hold true as well for ICI-associated metabolic and nutritional toxicities. Although the combination immunotherapies, mainly nivolumab + ipilimumab, have been evaluated in several cancers (28), their potential toxicity should always be balanced against their benefits. Of note, a variety of signals were generated with varied reporting frequencies for different ICI strategies and several of them were quantified at the first time. Considering that safety data regarding metabolic and nutritional toxicities after receiving ICIs combination therapies was limited, this current study with enormous records at the national level provided a considerable number of signals for further analysis.

### Type 1 Diabetes Mellitus and Diabetic Ketoacidosis

Diabetes mellitus secondary to treatment with ICIs is a new entity (29). It has been reported that metabolic events are fundamentally linked with the induction of autoimmune type 1 diabetes mellitus (T1DM) in the context of a generalized expansion of the immune response (30). T1DM is immune-mediated and the activation of immunological pathways is expected to accelerate the disease process (31, 32). ICIs -triggered T1DM has been characterized as an approximately incidence of 0.1% in clinical trials (33). T1DM has been listed as a treatment-related adverse reaction in the prescribing labels for nivolumab, pembrolizumab, avelumab, and durvalumab (34). Fulminant diabetes, a subtype of T1DM and characterized by the very rapid disease progression, is of a major concern with usually high mortality (35, 36). We observed that T1DM could be followed by anti-PD-1, anti-PD-L1 and anti-CALT-4 monotherapy, especially anti-PD-1. Two single-agent therapy, nivolumab and pembrolizumab, were detected with comparable signal values, corresponding to IC_025_/ROR_025_ of 3.65/13.37 and 3.53/12.26, respectively. Notably, the addition of CTLA-4 antibodies with PD-1/PD-L1 antibodies (nivolumab+ ipilimumab and pembrolizumab+ ipilimumab) could increase the risk, corresponding to IC_025_/ROR_025_ of 4.44/23.02 and 3.73/14.78, respectively. Additionally, another related event that needed to be on the alert is diabetic ketoacidosis, a usual clinical presentation for T1DM (37), which is a common cause for admission in Emergency Department and potentially life threatening. A previous review suggested clinicians to be aware of diabetic ketoacidosis during anti-PD-1 therapy (38). Our findings indicated that not only anti-PD-1 therapy but also the combination of ipilimumab plus nivolumab (IC_025_/ROR_025 _= 2.89/7.70) or plus pembrolizumab (IC_025_/ROR_025 _= 2.15/5.21) seemed to further reinforce the toxicity, which needed more attention. Periodic measurement of blood is strongly recommended in clinical practice for ensuring an early intervention to prevent these severe complications.

### Hypothyroidism and Hyperthyroidism

Both hypothyroidism and hyperthyroidism are common clinical manifestations of thyroid dysfunctions following ICIs administration. A meta-analysis showed that the incidence of hypo- and/or hyperthyroidism might be up to 5.6% (39). It supported that thyroid dysfunction appeared to be more frequently associated with PD-1, rather than CTLA-4 (40). In our study, for hypothyroidism, the strongest signal was detected for nivolumab monotherapy (IC_025_/ROR_025 _= 4.16/18.93), followed by nivolumab+ ipilimumab therapy (IC_025_/ROR_025 _= 3.56/12.54), which was inconsistent with the results from two clinical trials suggesting that patients receiving a combination of nivolumab and ipilimumab therapy had the highest chance of developing hypothyroidism (41, 42). However, regarding hyperthyroidism, nivolumab+ ipilimumab therapy was found associated with the greatest signal (IC_025_/ROR_025 _= 4.30/20.72), followed by pembrolizumab therapy (IC_025_/ROR_025 _= 3.47/11.66). This was supported by a prior study that predicted the highest incidence of hyperthyroidism for nivolumab plus ipilimumab therapy, followed by anti-PD-1 therapy (43). Moreover, it was notable that combining pembrolizumab with ipilimumab expressed less these toxicities, corresponding to an IC_025_/ROR_025_ of 1.87/4.47 and 2.41/6.35, respectively.

### Respiratory Failure and Acute Respiratory Failure

Despite uncommon, respiratory failure and acute respiratory failure toxicities have emerged in patients receiving ICIs (44–46). Acute respiratory failure is considered the leading cause of ICU admission in immunocompromised patients accompanied by high morbidity and mortality. Novel cancer therapies, including ICIs and chimeric antigen receptor T-cell therapy (CAR T-cell), have contributed to this problem (47). With the widespread use of ICIs, treatment-related or disease-related acute respiratory failure has also been observed, and some of the cases were even fatal. Currently, most cases reported acute respiratory failure AEs were derived from single-agent therapies. Our study first characterized their occurrences in combination therapies. In our study, highly reported frequencies of acute respiratory failure were observed in three single-agent therapies, including nivolumab (IC_025_/ROR_025 _= 2.27/5.03), pembrolizumab (IC_025_/ROR_025 _= 1.72/3.50), and atezolizumab (IC_025_/ROR_025 _= 1.98/4.37), as well as combination therapy nivolumab+ ipilimumab (IC_025_/ROR_025 _= 1.86/3.88). Further investigation is needed to reveal the underlying mechanisms.

### Hypercalcaemia, Hyponatraemia, Hypokalaemia, and Hypoalbuminaemia

There is limited data regarding the reporting of hypercalcaemia, hyponatraemia, hypokalaemia, and hypoalbuminaemia in a real-world population of patients receiving ICIs (11, 48, 49) and the safety profile of these disorders in patients receiving ICIs have not been characterized. It has been reported that endocrinopathies such as such as hypothyroidism, adrenalitis and hypophysitis can be associated with hyponatremia in patients receiving ICIs (9–11). A recent retrospective observational study also found that hyponatremia is very common in patients receiving ICIs and any-grade hyponatremia was significantly more common in patients receiving combination therapy (9). Noteworthily, in this current study, hyponatraemia events were disproportionately reported in most ICI regimens and the strongest signal was detected for nivolumab+ ipilimumab (IC_025_/ROR_025 _= 2.65/6.49). Regarding hypercalcaemia, disproportionality association were detected with several ICI strategies and the strongest signal emerged for nivolumab (IC_025_/ROR_025 _= 2.49/5.88), followed by nivolumab+ ipilimumab (IC_025_/ROR_025 _= 2.33/5.02). Interestingly, we did not detect an association between ICIs and hypocalcemia which was inconsistent with the result from a prior meta study showing that treatment with PD-1 inhibitors is linked with an augmented risk of developing hypocalcemia (12). Actually, Seethapathy H et al.’s research result also found no significant association between ICIs and hypocalcemia (9). Additionally, in our study, no disproportionate association between hypophosphatemia and any ICI strategies was observed which differed from the results of Seethapathy H et al. (9). Significant over-reporting frequencies of hypokalaemia and hypoalbuminaemia were also observed in several ICI treatment strategies. Nivolumab+ ipilimumab was detected to hold the strongest association with hypokalaemia (IC_025_/ROR_025 _= 1.11/2.28) and pembrolizumab shown the strongest association with hypoalbuminaemia (IC_025_/ROR_025 _= 2.18/4.96). Indeed, both hypercalcaemia (50) and hyponatremia (51) have been reported to be a common metabolic disorder in cancer patients, associated with poor prognoses and even death. Additionally, hypokalemia has also been suggested to be a risk factor for sudden cardiac death (52). If not recognized early and left uncorrected, it could lead to serious consequences and even death. Given the increasing and expanding use of ICIs in clinical practice, it is imperative that clinicians should be aware of the high risk of these abnormalities. Our results underlined the importance of a systematic approach to the investigation of these diseases in the recipients of ICIs. Moreover, periodic and closely monitoring are also recommended to maintain normal blood calcium, sodium, potassium and protein concentration. Additional further research is required to reveal the mechanisms driving electrolyte abnormalities in patients on ICIs.

There are several limitations in our study that should be acknowledged. Firstly, the bias derived from the nature of the self-reporting database including both over- and under-reporting was inevitable. Secondly, variable values that might contribute to a better analysis regarding the response rate and durability of the response were missing considerably in the FAERS database. Thirdly, most of the reporting data in FAERS come from America and Europe countries, which might lead to geographic bias of the results. The question should be addressed by combining FAERS with other sources of spontaneous reporting data from Asian countries. We hope our study could inspire future study further exploring this issue. Fourthly, despite the efficiency and popularity of disproportionality methods in signal detection, their shortcomings in dealing with confounding like masking effect and co-prescription should also be properly addressed (16). It is worth noting that signals detected in our study do not mean a causal relationship, rather, they should be considered as initial warnings that deserve further investigation using other data sources. Despite the limitations aforementioned, our analysis based on nearly thirty million records from real-world clinical practice may provide important clues for conducting relevant clinical studies better in the future.

## Conclusion

A wide variety of metabolic and nutritional signals emerged with varied reporting frequencies for different ICI strategies in the present study. A comprehensive understanding of their occurrences for different ICI regimens is needed to improve cancer survivorship in affected populations. In addition, periodic measurement of related blood biochemical parameters is also vital to prevent potentially serious complications.

## Data Availability Statement

Publicly available datasets were analyzed in this study. This data can be found here: https://fis.fda.gov/extensions/FPD-QDE-FAERS/FPD-QDE-FAERS.html.

## Author Contributions

Conception and design: YHZ, XFY, and FYH. Administrative support: JH and YC. Acquisition or review of data: JFX, XJG,XZ, and YZ. Data analysis and interpretation: YHZ and FYH. Manuscript writing: YHZ. Manuscript reviewing and revising: YHZ, XFY, FYH, and YC. All authors contributed to the article and approved the submitted version.

## Funding

This study was supported by the National Nature Science Foundation of China (No. 82073671), the Leading Talents of Public Health in Shanghai (No. GWV-10.2-XD22), the Shanghai Municipal Commission of Health and Family Planning Fund for Excellent Young Scholars (No. 2018YQ47), and the Excellent Young Scholars of public health in Shanghai (No. GWV-10.2-YQ33), three-year Action Program of Shanghai Municipality for Strengthening the Construction of Public Health System (GWV-10.1-XK05) Big Data and Artificial Intelligence Application, and Military Key Discipline Construction Project (Health Service–Naval Health Service Organization and Command) (No.03).

## Conflict of Interest

The authors declare that the research was conducted in the absence of any commercial or financial relationships that could be construed as a potential conflict of interest.

## Publisher’s Note

All claims expressed in this article are solely those of the authors and do not necessarily represent those of their affiliated organizations, or those of the publisher, the editors and the reviewers. Any product that may be evaluated in this article, or claim that may be made by its manufacturer, is not guaranteed or endorsed by the publisher.

## Abbreviations

AEs, adverse events; CTLA-4, Cytotoxic T-lymphocyte-associated protein; FAERS, FDA adverse event reporting system; FDA, Food and Drug Administration; HLGT, high-level group term; HLT, high-level term; IC, information component; ICIs, immune checkpoint inhibitors; irAEs, immune-related adverse events; MedDRA, Medical Dictionary for Regulatory Activities; MNDs, metabolic and nutritional disorders; N, number of records; PD-1, Programmed cell death-1; PD-L1, Programmed cell death-ligand 1; PT, preferred term; ROR, reporting odds ratio; SOC, system organ class; T1DM, type 1 diabetes mellitus.
