# Supplementary material for: Metabolic and Nutritional Disorders Following the Administration of Immune Checkpoint Inhibitors: A Pharmacovigilance Study
Source: Front Endocrinol (Lausanne). 2022 Jan 25;12:809063. doi: 10.3389/fendo.2021.809063 (PMC8821653; doi:10.3389/fendo.2021.809063)
Supplement: Supplementary file 1 [file Table_1.docx]

**Table S1.** Metabolic and nutritional events grouping as 14 broad entities according to MedDRA 22.0

| **Code** | **Name** | **Level** |
| --- | --- | --- |
| 10027433 | Metabolism and nutrition disorders | SOC |
| 10000485 | Acid-base disorders | HLGT |
| 10000546 | Metabolism disorders NEC | HLGT |
| 10003018 | Appetite and general nutritional disorders | HLGT |
| 10012653 | Diabetic complications | HLGT |
| 10013296 | Bone, calcium, magnesium and phosphorus metabolism disorders | HLGT |
| 10013317 | Lipid metabolism disorders | HLGT |
| 10014412 | Electrolyte and fluid balance conditions | HLGT |
| 10016950 | Food intolerance syndromes | HLGT |
| 10018424 | Glucose metabolism disorders (incl diabetes mellitus) | HLGT |
| 10021605 | Inborn errors of metabolism | HLGT |
| 10022958 | Iron and trace metal metabolism disorders | HLGT |
| 10037008 | Protein and amino acid metabolism disorders NEC | HLGT |
| 10037546 | Purine and pyrimidine metabolism disorders | HLGT |
| 10047635 | Vitamin related disorders | HLGT |

* SOC: system organ class; HLGT: high-level group term.
